# Beyond the fruit: phenolic composition, cytotoxicity, antioxidant, and multi-enzyme inhibitory activity of twig and leaf extracts from four understudied Turkish *Rosa* species

**DOI:** 10.1039/d6ra07053a

**Published:** 2026-09-11

**Authors:** Hajar Salehi, Milena Terzic, Gunes Ak, Gokhan Zengin, Łukasz Świątek, Kinga Salwa, Ismail Yapıcı, Ilhami Gulcin, Evren Yildiztugay, Luigi Lucini

**Affiliations:** a Department for Sustainable Food Process, Università Cattolica del Sacro Cuore Piacenza Italy hajar.salehi@unicatt.it; b Faculty of Technology Novi Sad, University of Novi Sad Novi Sad Serbia; c Department of Biology, Science Faculty, Selcuk University Konya Turkey gokhanzengin@selcuk.edu.tr; d Department of Virology with Viral Diagnostics Laboratory, Medical University of Lublin Lublin Poland; e Gümüşhane University Central Research Laboratory Gümüşhane Turkey; f Department of Chemistry, Science Faculty, Ataturk University Erzurum Turkey; g Rectorate of Agri İbrahim Çeçen University 04100 Agri Turkey; h Department of Biotechnology, Science Faculty, Selcuk University Konya Turkey; i Institute of Bioimaging and Complex Biological Systems (IBSBC), National Research Council (CNR) Milan Italy

## Abstract

The phytochemical profile and multi-target bioactivity of four Turkish *Rosa* species, *R. canina*, *R. hemisphaerica*, *R. horrida*, and *R. pulverulenta*, were evaluated across vegetative organs (twigs and leaves) and three solvent polarities (ethyl acetate (EA), methanol (MeOH), water), in total 24 extracts. Untargeted Ultra-High-Performance Liquid Chromatography-Quadrupole Time-of-Flight Mass Spectrometry (UHPLC-QTOF-MS) metabolomics, along with total phenolic (TPC) and flavonoid (TFC) content, six antioxidant assays and inhibition of seven enzymes were determined. Twig EA extracts displayed unexpectedly high TPC (83–105 mg gallic acid equivalent (GAE) per g) and TFC (35–47 mg rutin equivalent (RE) per g), indicative of selective aglycone flavonoid enrichment. The phenolic profiles identified 337 putative compounds, of which 60.84% belonged to the flavonoid class. The semi-quantitative analysis also indicates that methanol and water extracts had higher quantities of glycosylated flavonoids and phenolic acids, while EA extracts were richer in lignans, especially *R. horrida*-leaf-EA (1743.25 µg SE per g DW), and polyphenols. In enzyme inhibition assays, EA extracts demonstrated consistent multi-target activity: AChE inhibition (up to 2.49 mg galantamine equivalent (GALAE) per g), BChE inhibition (up to 4.44 mg GALAE per g), tyrosinase inhibition (up to 63.58 mg kojic acid equivalent (KAE) per g), α-amylase inhibition (up to 0.72 mmol acarbose equivalent ACAE per g), and carbonic anhydrase inhibition (CA I IC_50_ as low as 12.17 µg mL^−1^). The *R. hemisphaerica*-twig-EA extract showed highly selective cytotoxic activity against A375 cells, with a 50% cytotoxic concentration (CC50) of 3.22 µg mL^−1^ and an SI of 73.95, compared to VERO cells. This is the first report of carbonic anhydrase inhibition, BChE-selective activity, cytotoxicity, and comprehensive enzyme-inhibitory data for *R. hemisphaerica*, *R. horrida*, and *R. pulverulenta*. These findings reframe the phytopharmacological potential of Turkish *Rosa* species beyond their traditionally studied fruit.

## Abstract 2

This is the first report of carbonic anhydrase inhibition, BChE-selective activity, cytotoxicity, and comprehensive enzyme-inhibitory data for *R. hemisphaerica*, *R. horrida*, and *R. pulverulenta*.

## Graphical abstract

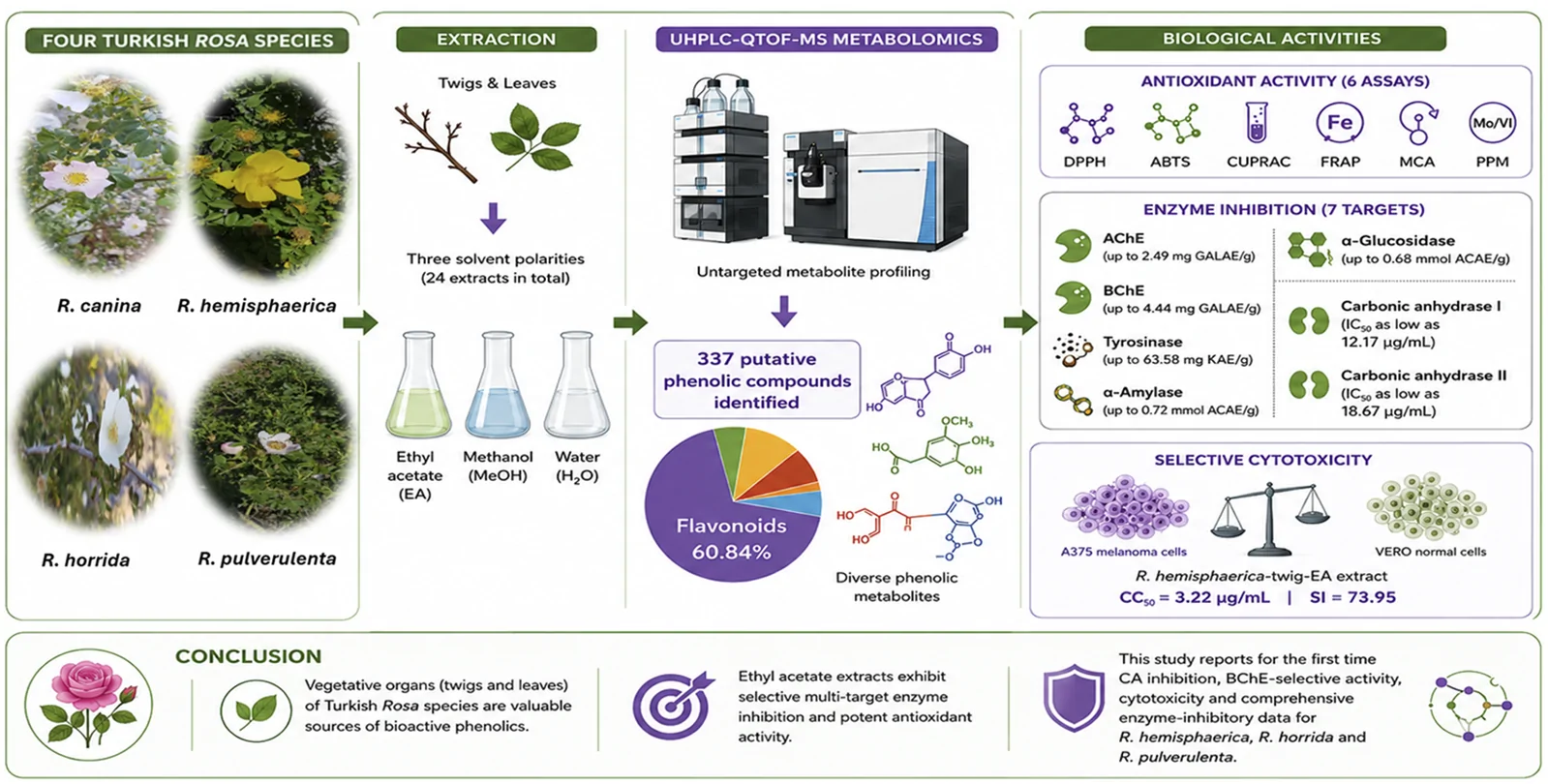

## 1. Introduction

In recent decades, natural products have been recognized as the source of most active ingredients used as raw materials for producing medicines and have attracted worldwide interest due to their protective effects against oxidative stress and several diseases in humans. The genus *Rosa* belongs to the Rosaceae family, and is an ornamental and a precious herb with modern pharmacological importance, which is used in medicines. There are about 18 000 cultivars and 200 species of the genus *Rosa* worldwide. Different organs of the plant, including both vegetative and generative parts, are rich in secondary metabolites. To date, phytochemical screenings have reported over 300 secondary metabolites, including 61 flavonoids, 83 triterpenes, 75 tannins, and 10 phenolic acids.^1^ These compounds have been documented to be involved in a wide range of biological activities and nutritional functions, such as antioxidant, anti-inflammatory, antimicrobial, and enzyme-inhibitory effects.^1–3^ For instance, compounds such as quercetin, kaempferol, ellagic acid, gallic acid, ursolic acid, are reported to be inhibitors of acetylcholinesterase (AChE), butyrylcholinesterase (BChE), tyrosinase, α-amylase, α-glucosidase, and carbonic anhydrase (CA).^1,3^

Regarding the genus *Rosa*, there are several reports on its chemical composition, but there is limited understanding of its bioactivity and mechanism of action. Furthermore, the bioactivities of *Rosa* plants are characterized mainly by focusing on a single species or on a specific organ, especially flower tissues. For instance, reports on bioactivities are largely derived from the fruits of *R. canina*. Recent systematic literature has identified a lack of data on remaining species, which limits the pharmacological potential of the genus.^1,2^ Turkey is considered one of the most important *Rosa* germplasm centers with around 30 native species. Among them, *R. hemisphaerica* Herrm., *R. horrida* Fisch., and *R. pulverulenta* M.Bieb., have been reported in Anatolian ethnobotanical surveys as treatment for inflammatory, gastrointestinal, and febrile conditions,^4,5^ yet to our knowledge, no pharmacological data are present for these species.

On the other hand, the available reports on *R. canina* derive mainly from the fruit, while other organs, including leaves and twigs, have been excluded. However, there is evidence that vegetative tissues may accumulate various polyphenolic compounds, such as flavonoid aglycones (catechin, epicatechin *etc.*), hydroxycinnamic acid derivatives (neochlorogenic acid, caffeic acid, ferulic acid, rosmarinic acid *etc.*) and tannins that are less present in polar extracts of fruits.^6,7^ Kubczak *et al.* reported that leaves and twigs of *R. canina* are rich in ellagic acid, myricetin, procyanidins, and quercetin.^6^

There are several reports on the antioxidant activity of extracts from certain *Rosa* species.^3,8–10^ For example, the high antioxidant potential of *R. canina* and the moderate activity of *R. arvensis* extracts were determined against DPPH, nitric oxide, superoxide anion, and hydroxyl radical, as well as their reducing power and inhibition of lipid peroxidation.^9^ In addition, a strong correlation has been observed between total phenolic content and the potential radical-scavenging and reducing activities of *Rosa* extracts.^8^ Despite the claim that enzyme inhibition of *Rosa* extracts is attributed to polyphenol, there are few reports on the enzyme inhibitory activity of the species native to Turkey. For instance, AChE and BChE inhibition has been reported for *R. pisiformis*^11^ and *R. pimpinellifolia*;^12^ antidiabetic enzyme inhibition for *R. acicularis* and *R. pisiformis*;^11,13^ tyrosinase inhibition for *R. damascena*^14^ and *R. canina* root.^15^ Furthermore, in most cases, screening has been conducted at the species level, with no cross-species or cross-organ comparisons. Moreover, the available studies have mainly used polar solvents for extraction, such as ethanol and water, thereby preventing the lipophilic phenolic fraction from being characterized. For instance, aglycone flavonoids, which are more readily extracted into apolar fractions, have been reported to inhibit hydrophobic enzyme active sites more efficiently than their glycosidic counterparts.^3,16–18^

In this study, we investigated the phytochemical characterization of four Turkish *Rosa* species (*R. canina*, *R. hemisphaerica*, *R. horrida*, *R. pulverulenta*) across two vegetative organs (leaves and twigs) using three solvents of varying polarity. Specifically, an untargeted metabolomics analysis was performed to identify a wide range of bioactive compounds. Afterward, the antioxidant and enzyme inhibitory activities of the extracts were monitored using various and complementary methods. In addition, the cytotoxic effects of the extracts were evaluated against several cancer cell lines (H1HeLa, MDA-MB-231, A375) and a non-cancer cell line (VERO).

## 2. Materials and methods

### 2.1. Plant collection

Plant samples were collected in 2022 from Konya, Turkey. The location information is given below. Dr Evren Yildiztugay officially carried out the botanical identification. A reference specimen was placed at the Selcuk University herbarium.

#### 2.1.1. *Rosa canina* L.

Bozkır, Konya, Akçapınar around, forest entrance, 1230 m. Voucher number: EY-3102.

#### 2.1.2. *Rosa hemisphaerica* J. Herrm.

Bozkır, Konya, entrance to Yalnızca location, roadside, 1500 m. Voucher number: EY-3104.

#### 2.1.3. *Rosa pulverulenta* Bieb.

Bozkır, Konya, Dipsiz Lake around, rocky habitat, 1700 m. Voucher number: EY-3114.

#### 2.1.4. *Rosa horrida* Fisch.

Bilecik location, Konya, Türkiye; roadside at the exit of Bilecik, 1100 m. Voucher number: EY-3119.

The twigs and leaves were separated immediately after collection and allowed to air-dry at room temperature in a well-ventilated, shady area. The plant material was pulverized into a fine powder once it had dried completely. The powdered samples were kept under constant conditions in light-proof containers to maintain chemical stability and prevent spoiling.

### 2.2. Plant extract preparation

To separate bioactive chemicals, three solvents of varying polarities (distilled water, methanol, and ethyl acetate) were used. Ten grams of dried plant material were mixed with two hundred milliliters of each solvent. Depending on the type of solvent used, the extraction process was different. The plant material (5 g) was macerated by soaking it in organic solvents (200 mL methanol or ethyl acetate) for a whole day at room temperature. Instead, a 15 minutes hot-water infusion was used for water-based extraction. Following extraction, the organic solvent extracts were concentrated under reduced pressure using a rotary evaporator, and the aqueous extract was freeze-dried. Extraction yields are given in Table 1.

**Table 1:** Extraction yields (%), the total phenolic and flavonoid content in the tested extracts^a^

*Rosa* species	Parts	Extracts	Extraction yields (%)	TPC (mg GAE per g)^b^	TFC (mg RE per g)^c^
*Rosa canina*	Twigs	EA	1.26	83.58 ± 1.77^g^	35.52 ± 0.71^h^
		MeOH	12.44	110.42 ± 0.48^abc^	24.76 ± 0.30^m^
		Water	5.62	111.35 ± 1.27^ab^	21.97 ± 0.25^n^
	Leaves	EA	1.10	39.68 ± 0.82^h^	8.70 ± 0.33^r^
		MeOH	30.89	110.39 ± 0.54^abc^	55.92 ± 1.21^b^
		Water	14.91	112.95 ± 1.06^a^	50.05 ± 0.30^de^
*Rosa hemispherica*	Twigs	EA	2.42	98.69 ± 0.62^f^	12.90 ± 0.43^q^
		MeOH	11.97	109.35 ± 0.94^abcde^	14.56 ± 0.17^p^
		Water	7.44	105.86 ± 5.47^cde^	12.65 ± 0.37^q^
	Leaves	EA	2.78	21.09 ± 0.13^j^	11.61 ± 0.49^q^
		MeOH	27.88	110.83 ± 0.88^ab^	52.24 ± 0.54^c^
		Water	10.77	107.66 ± 1.61^bcde^	33.99 ± 0.38^i^
*Rosa horrida*	Twigs	EA	1.66	105.36 ± 1.10^de^	27.36 ± 0.64^k^
		MeOH	13.09	110.37 ± 0.14^abc^	24.98 ± 0.05^lm^
		Water	7.70	110.87 ± 1.35^ab^	21.40 ± 0.36^n^
	Leaves	EA	2.23	40.38 ± 0.89^h^	30.65 ± 0.30^j^
		MeOH	30.75	109.59 ± 2.04^abcd^	57.82 ± 0.08^a^
		Water	8.12	109.95 ± 0.90^abcd^	50.70 ± 0.34^d^
*Rosa pulveranta*	Twigs	EA	1.37	83.00 ± 1.48^g^	46.70 ± 0.68^g^
		MeOH	13.89	107.10 ± 1.21^bcde^	31.55 ± 0.21^j^
		Water	6.33	108.47 ± 1.50^abcde^	26.37 ± 0.30^kl^
	Leaves	EA	1.64	30.89 ± 0.30^i^	17.26 ± 0.22^o^
		MeOH	21.04	104.59 ± 0.68^e^	48.53 ± 0.22^f^
		Water	11.13	97.30 ± 0.54^f^	48.73 ± 0.53^ef^

a Values are means ± SD of three measurements. Different letters indicate significant differences between the tested extracts (*p* < 0.05).

b mg Gallic acid equivalents per gram of extract.

c mg Rutin equivalents per gram of extract.

### 2.3. Total phenolic and flavonoid contents

Total phenolic^19^ and total flavonoid^20^ contents in the extracts were determined using validated colorimetric methods, following established procedures. Calibration curves were prepared using gallic acid for phenolics and rutin for flavonoids, enabling the results to be expressed as GAE and RE per gram, respectively.

### 2.4. Untargeted UHPLC-HRMS metabolomics analysis

To investigate the chemical profile of *Rosa* samples, an untargeted metabolomics analysis was conducted using ultra-high-performance liquid chromatography coupled with electrospray ionization quadrupole time-of-flight mass spectrometry (UHPLC-ESI/QTOF-MS). For sample preparation, the same extracts as previously described in Section 2.1 were resuspended (1 : 10, w/v) in distilled water, ethyl acetate, and 0.1% formic acid in 80% aqueous methanol. The extracts were then centrifuged at 7197×*g* at 4 °C for 15 min and filtered through 0.22 µm syringe filters. 6 µL of each extract was injected into a 1290 liquid chromatograph (column: 2.1 × 100 mm, 1.9 µm) coupled with a G6550 mass spectrometer detector. The mobile phase included a binary mixture of water (A) and acetonitrile (B), both acidified with 0.1% (v/v) formic acid. A gradient elution was applied, where solvent A decreased from 94% to 6% over 32 min at a flow rate of 200 µL min^−1^. The mass spectrometry data were acquired in positive ion mode using SCAN acquisition, covering an *m*/*z* range of 100–1200 with a resolution of 30 000 (FWHM).

Data annotation was processed using Agilent Profinder B.15.1 software. Only compounds detected in at least 75% of replicates were retained for further analysis. Compounds were identified using the find-by-formula algorithm by matching features against the Phenol-Explorer database (http://phenol-explorer.eu), with a confidence level of 2 (COSMOS metabolomics standards) assigned for compound annotation.^21,22^

The semi-quantitative analysis of bioactive compounds was performed by utilizing standard curves of sesamin for lignans (*y* = 229 159*x*, *R*_2_ = 0.993); ferulic acid for phenolic acids (*y* = 457 774*x*, *R*_2_ = 0.999); cyanidin for anthocyanins (*y* = 599 208*x*, *R*_2_ = 0.998); tyrosol for low molecular weight compounds (abbreviated as LMW, including coumarins, tyrosols, phenolic aldehydes and alkylphenols) (*y* = 258 779*x*, *R*_2_ = 0.986); (+)-catechin for flavanols (*y* = 790 261*x*, *R*_2_ = 0.997); quercetin for flavonols (*y* = 1 090 960*x*, *R*_2_ = 0.987); and luteolin for flavones and other flavonoids, including flavanones and isoflavonoids (*y* = 3 000 000*x*, *R*_2_ = 0.988); resveratrol for stilbenes (*y* = 897 032*x*, *R*_2_ = 0.991).

### 2.5. Biological activities (antioxidant and enzyme inhibition)

A set of *in vitro* assays was employed to evaluate antioxidant activity, following the protocol described by Grochowski *et al.*^23^ Reducing power was determined using the FRAP and CUPRAC methods, while radical scavenging capacity was assessed by DPPH and ABTS assays. The outcomes of these four tests were converted into Trolox equivalents and reported as mg TE per gram of dried extract. In addition, total antioxidant capacity was measured using the phosphomolybdenum (PPM) assay (mmol TE per g), and metal chelating activity was expressed as mg EDTA equivalents per gram (mg EDTAE per g).

Following established colorimetric methods,^23^ the extracts were evaluated for their ability to inhibit five key enzymes: acetylcholinesterase (AChE), butyrylcholinesterase (BChE), tyrosinase, α-amylase, and α-glucosidase. To ensure comparability among samples, inhibition results were expressed relative to appropriate reference compounds. Accordingly, cholinesterase inhibition was reported as mg GALAE per g, α-amylase and α-glucosidase inhibition as mmol ACAE per g, and tyrosinase inhibition as mg KAE per g.

Human carbonic anhydrase enzyme was purified by Sepharose 4 B-l-tyrosine sulfanilamide affinity column and characterized by SDS-PAGE as previously applied.^24^ The esterase activity of the carbonic anhydrase (CA) enzyme yields a yellow-colored aromatic *p*-nitrophenolate compound at a pH of around pH = 7.5. By taking advantage of this transformation, the esterase activity of CA enzymes can be determined spectrophotometrically. Various concentrations of the samples in 0.05 M pH = 7.4 Tris–SO_4_, 0.07 mM of *p*-nitrophenylacetate (in 1 : 25 acetone : water), and 20 µL of the enzyme were gently vortexed, and as soon the as the addition of the enzyme, the absorbance change at 348 nm was monitored by measuring minute intervals for three minutes. Control reactions and blank reactions were set up without inhibitors and enzymes. These steps were repeated until more than half of the enzyme activity was inhibited. All data were processed, and graphs were created using GraphPad Prism 8.0.2. IC_50_ value refers in this study to the concentration of an inhibitor needed to inhibit an enzyme activity response by 50%.

### 2.6. Cytotoxicity

The cytotoxicity of extracts from all tested *Rosa* species was evaluated *in vitro* against normal VERO (ATCC, CCL-81) cells and cancer-derived cell lines – A375 (ATCC, CRL-1619, malignant melanoma), H1HeLa (ATCC, CRL-1958, human cervical adenocarcinoma), and MDA-MB-231 (ATCC, HTB-26, human breast adenocarcinoma) – using a 3-(4,5-dimethylthiazol-2-yl)-2,5-diphenyltetrazolium bromide (MTT)-based protocol.

Media used for *in vitro* culturing included Dulbecco Modified Eagle Medium (DMEM, Corning, Tewksbury, MA, USA) for VERO and A375 cells, and Modified Eagle Medium (MEM, Corning) for other cell lines. Cell media used in the experiments were supplemented with antibiotics (Penicillin–Streptomycin Solution, Corning) and fetal bovine serum (FBS, Corning) – 10% (cell passaging) and 2% (cell maintenance and experiments). Phosphate-buffered saline (PBS) and trypsin were purchased from Corning, whereas 3-(4,5-dimethylthiazol-2-yl)-2,5-diphenyltetrazolium bromide was purchased from Sigma-Aldrich (St. Louis, MO, USA). Incubation was carried out in a 5% CO_2_ atmosphere at 37 °C (CO_2_ incubator, Panasonic Healthcare Co., Tokyo, Japan). Stock solutions of extracts were prepared by dissolving (50 mg mL^−1^) the extracts in cell culture grade DMSO (PanReac Applichem). Stock solutions of extracts were stored frozen (−23 °C) until used.

Cytotoxicity assessments were conducted in a BSL-2 laboratory environment, in accordance with previously outlined experimental protocols.^25^ Briefly, the cells were passaged into 96-well plates (Falcon, TC-treated, Corning) and, after overnight incubation, treated with serial dilutions of extract stock solutions for 72 h. Afterwards, the media was removed, cells were washed with PBS, and 10% of MTT solution (5 mg mL^−1^) in cell media was added, and the incubation continued for the next 3 h. Subsequently, the precipitated formazan crystals were dissolved, and the plates were left at 37 °C overnight. Finally, the Synergy H1 Multi-Mode Microplate Reader (BioTek Instruments, Inc., Winooski, Vermont, USA) with Gen5 software (ver. 3.09.07; BioTek Instruments, Inc.) was used to measure the absorbance (540 and 620 nm). Collected data was exported to GraphPad Prism, and the values of CC_50_ (cytotoxic concentrations; concentrations resulting in a 50% decrease of cellular viability) were calculated from dose–response curves (nonlinear regression model).

### 2.7. Statistical analysis

All experiments were conducted in triplicate, and variations among extract samples were evaluated for statistical significance. Data were analyzed using one-way ANOVA (GraphPad Prism, version 9.2), followed by Tukey's *post hoc* test for pairwise group comparisons. A *p*-value below 0.05 was regarded as statistically significant.

The spectra extracted from UHPLC-QTOF-MS analysis were aligned and normalized using Mass Professional B.15.1 software from Agilent Technologies. The samples were log 2-transformed, then normalized and baselined to the median abundance across all samples. Afterward, an unsupervised hierarchical cluster analysis (HCA) was performed using Euclidean distance, based on fold-change heatmaps. To evaluate the impact of various factors in this study, including species, tissue, solvent, and their interactions, on the metabolomic profile, a supervised analysis of variance multiblock orthogonal partial least squares discriminant analysis (AMOPLS) was performed in R (version 4.2.3) using the rAMOPLS package. The robustness and significance of the model were assessed through 100 permutation tests. Model interpretation was evaluated using the key metrics, including relative sum of squares (RSS), *p*-values, and principal predictive components. Variables Importance in Projection (VIP^2^) scores were calculated to rank the most discriminating compounds that contribute most to sample separation.

In addition, one-way and two-way analyses of variance (ANOVA) were conducted at a significant level of *p* < 0.05 on semi-quantitative data. The comparison among samples was determined using Tukey's *post hoc* test. The values are expressed as the mean ± standard deviation (SD) of three replicates. Pearson's correlation analysis and PCA biplot were performed in the R program.

## 3. Results and discussion

### 3.1. Untargeted phenolic profile and chemometric analysis

**Fig. 1:**
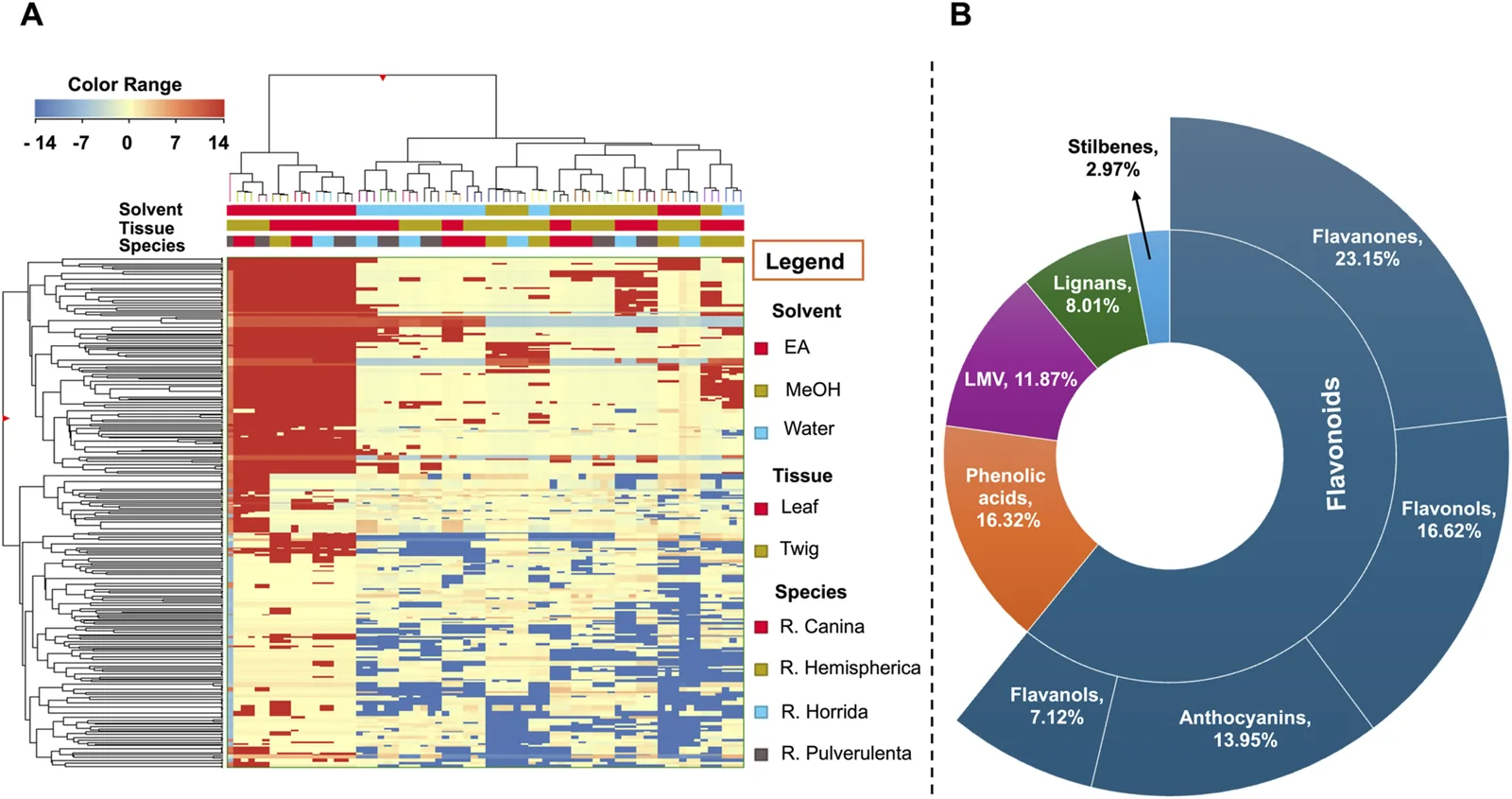
(A) Hierarchical analysis on the UHPLC-HRMS phenolic profile of *Rosa* samples, which was built based on the median abundance of all samples, considering the effect of species, tissue, and solvent. (B) Pie chart representing the distribution of bioactive compounds classified into different chemical classes.

**Fig. 2:**
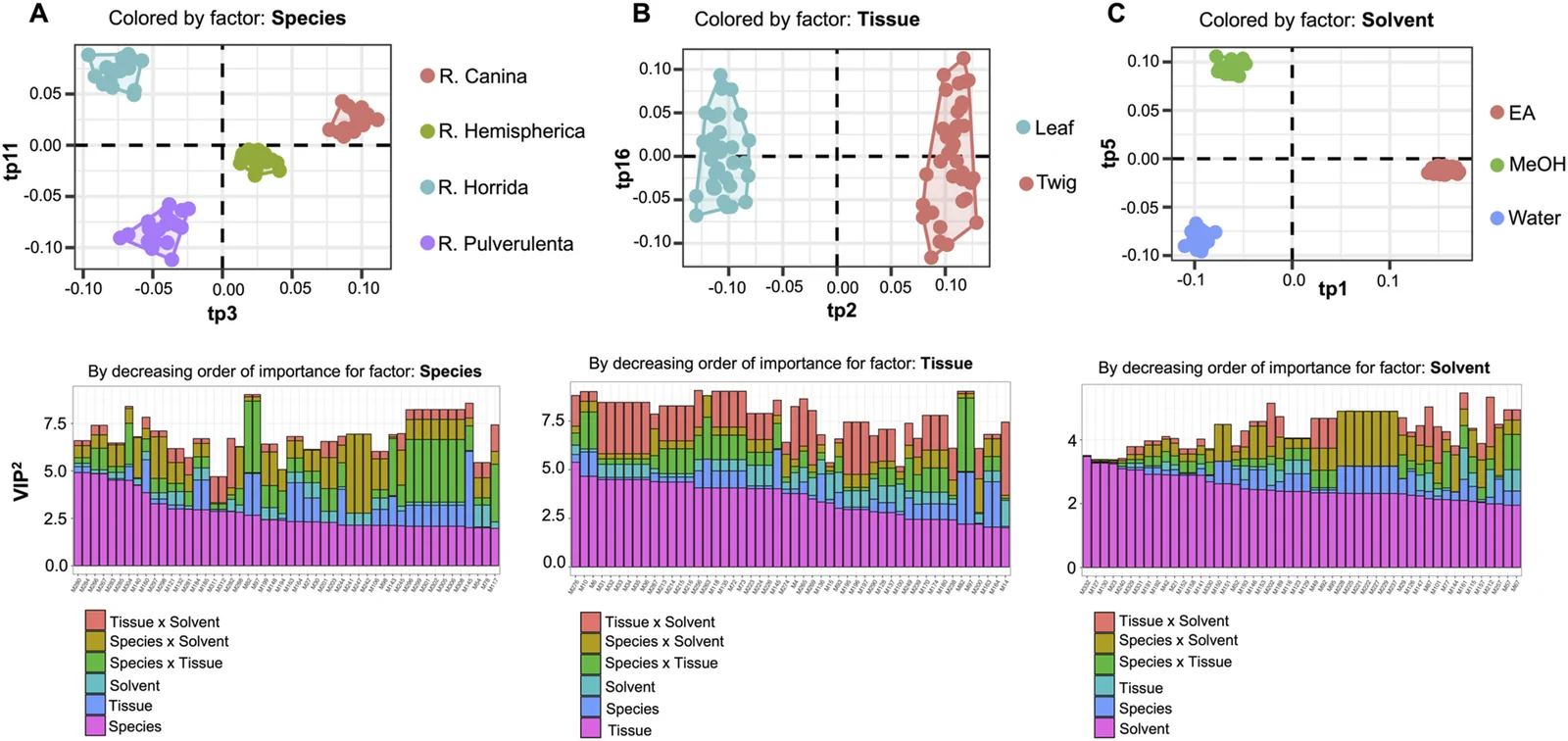
Supervised AMOPLS analysis score plots based on the phenolic profile, highlighting three key discriminant factors: species (A), tissue (B), and solvent (C), along with their corresponding total and factor-specific VIP_2_ values for the 50 compounds showing the most significant contribution to the factors. tp represents predictive components.

The impact of different variables (*i.e.*, tissue and solvent types) on the phenolic profile of *Rosa* species was evaluated through an untargeted metabolomics UHPLC-QTOF-MS approach. The putatively annotated compounds are reported in the SI (Table S1). Afterwards, the unsupervised HCA (based on fold-change values and normalized by the median) was performed to naively observe similarities and differences in the metabolic profile (Fig. 1A). The HCA showed that the most discriminating factor was solvent type, as two main clusters were observed: one for EA and the other for water and methanol; however, within the latter, water and methanol produced their own subcluster. Similarly, a previous study on the effect of different solvents on the metabolic profile of several Salsola species reported that water and methanol have similar metabolic profiles, which distinguishes them from the EA solvent.^26^ In addition, the PCA score plots from metabolomic analysis of three Asparagus species showed that samples were separated based on solvent polarity, in which, in all three species, the samples extracted with EA exhibited a distinct cluster from those extracted with methanol and chloroform.^27^ This suggests that the clustering based on solvent type is highly independent of the phytochemical composition of different species. To quantify the importance of each variable, including species, tissue, extraction, and their interaction, multivariate AMOPLS analysis was conducted (Fig. 2). The model, along with the predictive component values and corresponding scores, revealed that all factors and their interactions contributed to the observed variability; however, the extraction factor explained the largest contribution (28.6%) to the observed variability among others. The species factor and its interaction with extraction also accounted for 12.9% and 13.3% of total variation, respectively (Table 2). Despite being significant for both extraction and species variables, the results of the AMPOLS analysis here are inconsistent with the lower contribution of the extraction type over species type in the recent study that reported a higher contribution of the species variable (35%).^26^ This suggests that the metabolic composition of the *Rosa* genus across all four species might be more homogeneous at the species level than, for example, that of the *Salsola* genus. Furthermore, the significant species × extraction factor interaction indicates that the effect of solvent is not uniform across species, as certain classes of compounds are differentially enriched depending on the specific species–solvent combination, as supported by semi-quantitative analysis of bioactive compounds.

**Table 2:** Relative variability and block contributions of the AMOPLS analysis of *Rosa* phenolic data^a^

Effect Name	RSS%	RSS *p* value	R2Y *p* value	Block contributions							
				Tp1	Tp2	Tp3	Tp4	Tp5	Tp6	Tp7	To1
Species	12.9%	0.01	0.01	0.000	0.001	**0.892**	0.004	0.001	0.008	0.006	0.124
Tissue	8.2%	1.00	0.01	0.000	**0.990**	0.016	0.004	0.001	0.008	0.006	0.130
Solvent	28.6%	0.01	0.01	**0.997**	0.001	0.007	0.002	**0.989**	0.004	0.003	0.057
Species *x* tissue	8.6%	0.01	0.01	0.000	0.002	0.019	0.005	0.002	**0.947**	0.007	0.154
Species *x* solvent	13.3%	0.01	0.01	0.000	0.002	0.019	0.005	0.002	0.010	**0.959**	0154
Tissue *x* solvent	9.0%	0.01	0.01	0.000	0.002	0.018	**0.974**	0.002	0.009	0.007	0.145
Residuals	19.5%	0.01	0.01	0.001	0.003	0.029	0.007	0.003	0.015	0.011	0.236

a RSS: relative sum of squares, R2Y : fraction of the variation of Y explained by the model, tp1-7: predictive components, to: orthogonal component. The highest contribution for each component is reported in bold.

Regarding the different classes of bioactive compounds, the analysis showed that most of the compounds belonged to the flavonoid class (60.84%), including anthocyanins, flavanols, flavanones, and flavonols, indicating the prevalence of flavonoids (Fig. 1B). A recent review article reported a total of 61 compounds belonging to the flavonoids of the *Rosa* genus, where flavonols were found to be the main constituents, among others.^1^ Our results, however, identified a higher number of flavonoids (*n* = 205). In detail, anthocyanins (*n* = 47) were mostly represented by cyanidin, delphinidin, and pelargonidin derivatives, among others. The derivatives of theaflavin, procyanidin, and catechin constituted most of the compounds related to flavanols. In the same class, flavones that showed higher heterogeneity were mainly represented by luteolin and apigenin-related compounds. Similarly, kaempferol and quercetin derivatives constituted the flavonol subclass (*n* = 56). Our results are in agreement with previously reported HPLC and LC-MS/MS analyses that some compounds, such as quercetin, kaempferol, and isorhamnetin, are the main components.^28,29^ Other frequent classes were phenolic acids (16.32%), including hydroxybenzoic acids and hydroxycinnamic acids, lignans (8.01%), stilbenes (2.97%), and other polyphenols (11.87%).

Afterwards, the semi-quantitative analysis of variance (MANOVA) was performed to measure the levels of different bioactive compounds. The results for various compounds, including anthocyanins, flavanols, flavones, flavonols, lignans, phenolic acids, stilbenes, and other low molecular weight compounds, are provided in Table 3. The multivariate ANOVA analysis showed that in all cases, the variables, including species, tissue, extraction, and their interaction, are statistically significant. The statistical analysis also revealed that, in the flavonoid class, twig tissues generally had the highest levels of anthocyanins, flavanols, flavones, and flavonols. In contrast, leaf tissues were richer in lignans, phenolic acids, stilbenes, and other low molecular weight compounds. Similarly, the comparative analysis of phenolic compounds in *R. canina* showed that leaf tissues contain almost twice as much ellagic acid as twig tissues, and a higher content of cyanidin. In contrast, twig tissues were richer in procyanidins, which is consistent with the higher content of flavanol compounds in twig tissues across four species in our results. This tissue-dependent distribution of bioactive compounds may reflect either functional or biosynthetic origin.^30^ For instance, in woody plants, higher amounts of proanthocyanidins can increase defense mechanisms against pathogens, and their accumulation in the bark and cortical layers represents a constitutive chemical barrier. Similarly, the accumulation of phenolic acids and lignans in leaf tissue is in line with the active metabolic role of photosynthetic tissues, as reported in the tea plant (*Camellia sinensis*).^31^

**Table 3:** Semi-quantitative analysis of different classes of phenolic compounds in four *Rosa* species^a^

*Rosa* species	Parts	Extracts	Anthocyanins	Flavanols (µg CaE per g DW)	Flavones (µg LE per g DW)	Flavonols (µg QE per g DW)	Lignans (µg SE per g DW)	LMW and other polyphenols (µg TE per g DW)	Ph. acids (µg FE per g DW)	Stilbenes (µg RE per g DW)
*Rosa canina*	Twigs	EA	105.2 ± 24.65^fgh^	115.85 ± 5.8^ghij^	35.3 ± 1.35^defg^	83.75 ± 12.75^jkl^	107.75 ± 16.55^hi^	154.05 ± 18.2^l^	77.75 ± 0.65^l^	na
		MeOH	390.4 ± 13.25^cde^	304.35 ± 54.45^def^	66.55 ± 6.3^abcd^	252.85 ± 5.5^def^	364.8 ± 23.65^defg^	773 ± 62.25^b^	474.2 ± 36.25^defg^	9±2^efghi^
		Water	546.2 ± 27.1^bc^	451.05 ± 89.9^bc^	70.55 ± 14.5^abc^	248.6 ± 61.7^def^	285.95 ± 16.35^defgh^	299 ± 4.25^hijk^	229.95 ± 45.55^hijkl^	8.8±1^efghi^
	Leaves	EA	16.8 ± 6.85^h^	41.0 ± 29^hij^	8.55 ± 0.35^g^	36.55 ± 1.4^lm^	73.95 ± 22.05^i^	184.75 ± 20.2^kl^	105.2 ± 3.4^kl^	3.45 ± 0.55^hijk^
		MeOH	120.0 ± 28.35^fgh^	78.7 ± 9.55^hij^	74.7 ± 18.55^abc^	175.5 ± 3.5^ghi^	447.1 ± 18.95^cd^	420.85 ± 3.95^cdefg^	489.15 ± 11.3^def^	3.45 ± 0.1^hijk^
		Water	400.7 ± 30.35^c^	137.45 ± 21.25^ghij^	53.95 ± 7.5^bcde^	316.9 ± 23.5^bcd^	217.4 ± 36.65^efghi^	414 ± 13.05^defgh^	276.85 ± 52.35^hij^	13.5 ± 0.5^cde^
*Rosa hemisphaerica*	Twigs	EA	73.05 ± 2.50^gh^	412.25 ± 21.9^bcd^	22.3 ± 3.3^fg^	112 ± 72.35^jkl^	688.2 ± 4.6^b^	539.35 ± 60.05^c^	318.4 ± 20.25^ghi^	na
		MeOH	650.05 ± 18.15^ab^	402.9 ± 24.75^bcd^	75.5 ± 13^abc^	343.45 ± 13.35^bc^	619.65 ± 29.35^bc^	271.8 ± 28.65^ijkl^	628.25 ± 61.05^d^	18.3 ± 2.2^bc^
		Water	525.65 ± 172.3^bc^	129.1 ± 30.3^ghij^	64.75 ± 16.7^abcd^	312.6 ± 16.45^bcd^	372.6 ± 9.75^de^	315.85 ± 2.9^fghij^	462.45 ± 72.6^efg^	12.8 ± 0.6^cdef^
	Leaves	EA	106.15 ± 1.80^fgh^	46.4 ± 0.4^hij^	23.6 ± 1.7^efg^	185.5 ± 3.9^fgh^	644.05 ± 107.4^b^	2106.5 ± 36.75^a^	1561.15 ± 30.85^a^	6.1 ± 2.45^ghijk^
		MeOH	232.8 ± 12.55^ef^	123.8 ± 17.3^ghij^	50.1 ± 1.05^cdef^	131.35 ± 8.7^hijk^	398.85 ± 38.1^d^	500.9 ± 62.5^cd^	842.85 ± 12.85^c^	2.7 ± 0.6^ijk^
		Water	236.25 ± 26.75^def^	176.8 ± 6.6^fgh^	35.75 ± 2.05^defg^	114.25 ± 7.95^ijk^	365.55 ± 52.35^def^	433.1 ± 24.55^cdef^	508.95 ± 21.95^def^	19.95 ± 0.65^ab^
*Rosa horrida*	Twigs	EA	105.25 ± 36.55^fgh^	246.15 ± 26.6^efg^	21.1 ± 6.25^fg^	125.1 ± 25.4^hijk^	186.35 ± 3.1^ghi^	243.4 ± 27.65^jkl^	130.6 ± 2.85^jkl^	na
		MeOH	667.5 ± 20.3^ab^	412.25 ± 26.1^bcd^	81.8 ± 9.55^ab^	366 ± 8.7^b^	677.55 ± 6.7^b^	366.65 ± 26.35^efghi^	600.25 ± 70.5^de^	20.05 ± 1.65^ab^
		Water	561.35 ± 78.05^b^	601.2 ± 114.75^a^	89.3 ± 11^a^	291.35 ± 35.45^cde^	357.65 ± 76.35^defg^	236.45 ± 26^jkl^	252.55 ± 31.8^hijk^	6.7 ± 2.25^fghij^
	Leaves	EA	21.05 ± 4.05^h^	35.6 ± 1.45^ij^	18.65 ± 2.25^g^	65.8 ± 0.65^klm^	1743.25 ± 181.85^a^	488.1 ± 55.7^cd^	1022.3 ± 67.55^b^	10.35 ± 3.45^defg^
		MeOH	206.35 ± 33.05^fg^	52.45 ± 26.4^hij^	62.45 ± 4.4^abcd^	138.15 ± 12.7^hij^	444 ± 38.1^cd^	526.45 ± 36.2^cd^	483.55 ± 70.9^defg^	1.3 ± 0.3^jk^
		Water	741.35 ± 96.75^a^	295.3 ± 79.9^def^	68.85 ± 6.5^abc^	567.25 ± 31.2^a^	298.85 ± 26.9^defg^	677.85 ± 45.75^b^	516.05 ± 92.3^def^	9.2 ± 1.3^efgh^
*Rosa pulverulenta*	Twigs	EA	42.0 ± 7.30^h^	466.85 ± 23.5^ab^	23.55 ± 7.1^efg^	100.4 ± 36.3^hijk^	372.35 ± 26.4^de^	231.15 ± 46.55^jkl^	103.8 ± 3.05^kl^	na
		MeOH	620.4 ± 15.9^ab^	324.25 ± 73.7^cde^	91.4 ± 26.35^a^	374.3 ± 8.7^ab^	393.1 ± 22.05^de^	304.55 ± 10.65^ghij^	589.3 ± 133.85^de^	7.9 ± 0.35^efghi^
		Water	646.6 ± 89.95^ab^	68.8 ± 11.5^hij^	50.85 ± 6.5^bcdef^	309.05 ± 47.3^bcd^	190.2 ± 4.85^fghi^	309.3 ± 21.25^ghij^	201.05 ± 26.55^ijkl^	13.1 ± 0.7^cde^
	Leaves	EA	NA	15.9 ± 0.51^j^	12.8 ± 4.55^g^	6.45 ± 0.8^m^	417.9 ± 115.95^d^	234 ± 19.85^jkl^	376.1 ± 5.5^fgh^	6.3 ± 2.8^ghijk^
		MeOH	393.55 ± 27.95^cd^	96.6 ± 3.6^hij^	89.15 ± 5.5^a^	275.35 ± 11.3^cde^	311.15 ± 7.8^defg^	457.75 ± 47.35^cde^	942.95 ± 11.45^bc^	25.6 ± 0.6^a^
		Water	221.75 ± 7.85^fg^	156.05 ± 66.8^ghi^	65.15 ± 10^abcd^	237.6 ± 15^efg^	352.35 ± 42.85^defg^	680 ± 71.5^b^	352.2 ± 78.1^fghi^	15.65 ± 7.2^bcd^

a The data represents the mean of three technical replicates ± SD. Different letters indicate statistically significant differences (*p* < 0.05), determined by ANOVA followed by the *post hoc* test.

Considering the species factor, the accumulation of bioactive compounds followed a species-dependent trend. For instance, all flavonoid subclasses and lignans were higher in *R. horrida*, while *R. hemisphaerica* had higher phenolic acids and LMW. However, a heterogeneous trend was observed for the extraction factor. The semi-quantitative profiling of bioactive compounds revealed that methanol and water were the most productive solvents across species and tissues, yielding high levels of anthocyanins, flavonols, flavones, and phenolic acids. This can be attributed to the high solubility of the hydroxylated aglycone derivatives of phenolic compounds in water and alcohols.^32^ However, water extracts were richer in certain compound classes, such as anthocyanins, where *R. horrida* leaf water extract exhibited the highest anthocyanin content (741.35 µg CaE per g DW), and flavanols, where *R. horrida* twig water extract had the highest level (601.2 µg CaE per g DW), highlighting a higher degree of glycosylation of anthocyanins.^33^ On the other hand, ethyl acetate leaf extracts had a lower quantity of anthocyanins, flavanols, flavones, and flavonols across all four species. Moreover, several combinations exhibited a quantity of zero for some bioactive compounds, indicating that these compound classes are poorly soluble in low-polarity solvents. On the contrary, the distribution of lignans, LMW polyphenols, and phenolic acids showed a species- and tissue-specific trend in EA extracts. For instance, *R. horrida* leaf EA exhibited the highest lignan content (1743.25 µg SE per g DW), more than twice the next highest value, while *R. hemisphaerica* leaf EA resulted in the highest LMW polyphenol content (2106.5 µg TE per g DW) and the highest phenolic acid content (1561.15 µg FE per g DW). This suggests that in these two species, a pool of relatively non-polar phenolic compounds is concentrated specifically in leaf tissue and is selectively recovered by EA, compounds that would be diluted or masked in the more complex methanol and water extracts. This finding is pharmacologically relevant: lignans recovered in EA extracts are more likely to represent free aglycone forms, which generally display superior membrane permeability and enzyme-binding affinity relative to their glycosylated counterparts recovered in polar extracts. Among all compound classes, stilbenes had the lowest concentrations across all extracts, especially the EA solvent. The highest level of stilbene was found in *R. pulverulenta* leaf methanol (25.6 µg TE per g DW).

In addition, the bulk quantification of TPC and TFC of all 24 extracts was performed and is presented in Table 1. The results showed that the highest TPC content was observed in the MeOH and water extracts (≥97 mg GAE per g across all species and both tissues). These values were found to be higher than the ranges reported for ethanol extracts of fruits of previous *Rosa* species such as *R. canina*, *R. corymbifera*, *R. micrantha*, and *R. sempervirens*, which ranged from 10.89 to 67.85 mg GAE per g.^6,25^ However, in line with our results, previous studies reported a range of 138–141 mg GAE per g TPC for MeOH extraction of fruit and twig/leaf of *R. canina*.^6^ Our results showed that the vegetative organs of the investigated species contain a comparable quantity of phenolic compounds relative to fruit organs. It is worth mentioning that the extraction with EA, in general, resulted in lower TPC; however, the twig tissues in all species exhibited values ranging from 83–105 mg GAE per g, which is close those value of methanolic extractions. This is consistent with the previous report in *R. canina*, where the twig tissues were richer in procyanidins and catechin compared to leaves.^6^ In accordance with the semi-quantification of bioactive compounds, the highest TFC was observed in MeOH extractions up to 58 mg RE per g in *R. horrida* leaves, which is higher than values of 0.38–3.23 mg RE per g reported previously for fruits.^8,9^ This difference may arise because the leaves serve as the main source of production and accumulation of flavonoids.^1,34^ Furthermore, the twig extraction with EA in some species, such as *R. pulverulenta* and *R. canina*, had higher TFC compared to leaves, which was supported by higher content of flavanol, flavone, and flavonol classes.

Furthermore, to understand the effect of each variable on the phenolic profile, the VIP^2^ was calculated using the AMOPLS decomposition. The complete list of compounds along with their VIP^2^ scores is provided in Table S2. Considering the VIP^2^ value, it becomes apparent that compounds associated with flavonoids hold significant importance for tissue factor. Cyanidin 3-*O*-arabinoside, procyanidin, kaempferol 3-*O*-(6″-acetyl-galactoside) 7-*O*-rhamnoside, caffeoyl aspartic acid, and sinensetin were among the top-ranked compounds. The dominance of flavonoids as tissue discriminators is in line with the accumulation of glycosylated flavonoids in woody stem tissue. For instance, procyanidins have been reported as important components of bark and vascular tissue in the Rosaceae family.^6,35^ However, the species factor impacted a wider range of compounds, including polyphenols, phenolic acids, lignans, and flavonoids. Among them, Rosmadial, Delphinidin, and Kaempferol derivatives, sesamin, and gallic acids had the highest VIP score. The heterogeneity in VIP^2^ markers for species might reflect differences in the regulation of various biosynthetic branches of the phenylpropanoid pathway across the four species. For example, gallic acid, as the primary precursor of tannins, may indicate the inter-species variation consistent with species-specific regulation of the gallotanin and ellagitannin biosynthetic pathways in *Rosa*.^36,37^ The most important compounds discriminating the solvent factor were *p*-coumaroyl glycolic acid, matairesinol, phloridzin, xanthotoxin, oleuropein-aglycone, and a variety of flavonoids. Although these compounds belong to different structural classes, they are all moderately polar, and their recovery therefore varies markedly depending on the solvent used.

### 3.2. Antioxidant properties

The antioxidant capacity of all 24 extracts was evaluated using six complementary assays covering three mechanistically distinct pathways: DPPH and ABTS radical scavenging (mg TE per g),^38,39^ CUPRAC and FRAP reducing power (mg TE per g),^40,41^ and metal chelating activity (MCA; mg EDTAE per g),^42^ supplemented by the phosphomolybdenum assay (PPM; mmol TE per g^−1^) as a global reductive capacity estimate.^43^ Results are presented in Table 4.

**Table 4:** Antioxidant properties of the tested extracts^a^

*Rosa* species	Parts	Extracts	DPPH (mg TE g^−1^)^b^	ABTS (mg TE g^−1^)^b^	CUPRAC (mg TE g^−1^)^b^	FRAP (mg TE g^−1^)^b^	MCA (mg EDTAE/g)^c^	PPM (mmol TE g^−1^)^d^
*Rosa canina*	Twigs	EA	757.03 ± 18.38^i^	1779.28 ± 43.62^h^	520.59 ± 14.86^j^	440.18 ± 12.86^j^	15.32 ± 0.44^hij^	3.73 ± 0.23^defgh^
		MeOH	1001.72 ± 0.97^ab^	2278.02 ± 14.17^cd^	883.54 ± 13.46^d^	765.29 ± 6.46^de^	17.53 ± 0.20^efgh^	4.71 ± 0.24^ab^
		Water	939.53 ± 2.59^e^	2230.81 ± 4.62^cde^	785.95 ± 15.11^f^	736.46 ± 8.79^efg^	19.95 ± 5.12^cdef^	4.47 ± 0.05^bc^
	Leaves	EA	211.15 ± 1.80^k^	534.93 ± 2.19^j^	240.11 ± 3.37^l^	195.57 ± 5.63^l^	16.36 ± 0.64^fghi^	2.61 ± 0.18^lm^
		MeOH	1003.39 ± 1.32^a^	2452.39 ± 42.32^b^	961.48 ± 10.26^bc^	773.74 ± 9.06^de^	21.25 ± 0.09^bcd^	5.03 ± 0.35^a^
		Water	965.15 ± 2.45^d^	2083.60 ± 27.82^fg^	776.63 ± 2.16^fg^	796.87 ± 39.41^cd^	23.73 ± 0.25^ab^	3.76 ± 0.02^defgh^
*Rosa hemisphaerica*	Twigs	EA	827.62 ± 6.68^g^	1851.39 ± 40.67^h^	556.13 ± 2.45^ij^	515.32 ± 14.04^i^	14.33 ± 0.18^hij^	3.16 ± 0.15^ijk^
		MeOH	981.16 ± 1.08^abcd^	2323.37 ± 68.66^c^	855.07 ± 4.15^de^	725.70 ± 9.55^efg^	16.53 ± 1.32^fghi^	4.22 ± 0.20^bcd^
		Water	866.66 ± 4.24^f^	2143.76 ± 57.54^efg^	747.02 ± 7.85^g^	707.71 ± 6.07^fg^	21.94 ± 0.16^abc^	3.66 ± 0.16^efghi^
	Leaves	EA	90.67 ± 13.66^l^	259.30 ± 17.86^k^	177.12 ± 3.06^m^	165.04 ± 6.72^l^	9.28 ± 0.44^k^	2.48 ± 0.04^m^
		MeOH	994.11 ± 3.53^abc^	2517.73 ± 20.73^ab^	949.55 ± 20.82^c^	870.89 ± 31.75^b^	22.60 ± 0.07^abc^	5.06 ± 0.25^a^
		Water	864.79 ± 23.01^f^	1734.06 ± 11.90^h^	637.87 ± 6.99^h^	693.21 ± 19.76^g^	25.04 ± 0.12^a^	3.05 ± 0.07^jkl^
*Rosa horrida*	Twigs	EA	976.98 ± 2.63^bcd^	2067.66 ± 81.87^fg^	590.56 ± 6.87^i^	575.98 ± 3.75^h^	12.67 ± 0.68^jk^	4.13 ± 0.05^cde^
		MeOH	1005.02 ± 0.43^a^	2582.74 ± 33.52^a^	872.55 ± 27.44^de^	883.44 ± 50.48^b^	13.70 ± 0.50^ij^	4.46 ± 0.17^bc^
		Water	971.40 ± 0.93^cd^	2190.04 ± 74.68^def^	835.99 ± 9.80^e^	756.69 ± 4.92^def^	17.97 ± 0.65^defgh^	3.89 ± 0.12^defg^
	Leaves	EA	228.80 ± 7.05^k^	632.60 ± 26.87^j^	219.81 ± 3.56^l^	164.96 ± 3.18^l^	20.30 ± 0.20^bcde^	2.77 ± 0.02^klm^
		MeOH	1002.24 ± 1.13^a^	2303.39 ± 10.69^cd^	465.07 ± 10.54^k^	325.93 ± 13.94^k^	19.30 ± 0.88^cdefg^	3.99 ± 0.21^cdef^
		Water	982.12 ± 4.28^abcd^	2210.69 ± 34.34^cde^	1065.85 ± 15.85^a^	992.34 ± 16.63^a^	22.83 ± 0.09^abc^	3.36 ± 0.06^hij^
*Rosa pulverulenta*	Twigs	EA	789.10 ± 12.05^h^	1476.89 ± 22.24^i^	453.59 ± 9.08^k^	398.45 ± 9.60^j^	15.01 ± 0.20^hij^	3.56 ± 0.15^fghi^
		MeOH	1003.83 ± 1.77^a^	2499.27 ± 29.60^ab^	999.31 ± 11.29^b^	839.59 ± 13.58^bc^	16.25 ± 0.06^ghij^	5.00 ± 0.14^a^
		Water	938.05 ± 3.66^e^	2043.16 ± 15.88^g^	787.87 ± 16.35^f^	741.81 ± 0.52^defg^	20.00 ± 0.67^cdef^	4.20 ± 0.04^cd^
	Leaves	EA	23.43 ± 2.91^m^	211.29 ± 21.51^k^	223.20 ± 7.01^l^	139.01 ± 3.25^l^	13.27 ± 1.24^ij^	3.33 ± 0.12^hij^
		MeOH	966.02 ± 3.16^d^	1851.20 ± 46.12^h^	668.38 ± 13.48^h^	517.94 ± 23.57^i^	20.96 ± 0.07^bcde^	3.47 ± 0.21^ghij^
		Water	729.93 ± 10.39^j^	1554.32 ± 61.02^i^	525.69 ± 16.95^j^	527.71 ± 15.21^hi^	22.24 ± 0.23^abc^	2.77 ± 0.03^klm^

a Values are means ± SD of three measurements. Different letters indicate significant differences between the tested extracts (*p* < 0.05).

b mg Trolox equivalents per g of extract.

c mg EDTAE per g of extract.

d mmol Trolox equivalents per g of extract.

MeOH extracts demonstrated the highest or near-highest activity across DPPH, ABTS, CUPRAC, and FRAP for all species and organs, broadly paralleling their superior TPC values documented in Section 3.1. DPPH values for MeOH extracts reached the assay's effective saturation ceiling (∼1001–1005 mg TE per g across all species and tissues). This saturation phenomenon is well recognized and reflects the stoichiometric nature of the DPPH assay at extract concentrations exceeding the radical/antioxidant molar threshold,^38,44^ a consideration that reinforces the value of the multi-assay approach adopted here. Comparable DPPH saturation in MeOH extracts of *Rosa* vegetative material has been documented by Kubczak *et al.* for *R. canina* leaf and twig preparations,^6^ confirming that the polar phenolic fraction of vegetative *Rosa* tissue consistently reaches this ceiling. ABTS values, which measure both hydrophilic and lipophilic radical scavenging under aqueous conditions,^39^ were uniformly higher than corresponding DPPH values for all extracts, a pattern consistent with the broader mechanistic sensitivity of the ABTS assay and previously reported for *R. canina* and other *Rosa* species.^6,8^ Notably, the highest ABTS values recorded here, up to 2582.74 mg TE per g for *R. horrida* twig MeOH, substantially exceed the ABTS range of 165–340 µmol TE per g reported by Butkyevičiūtė *et al.* for ethanol rosehip extracts of ten *Rosa* species,^8^ confirming that the vegetative organs of the studied Turkish species harbor substantially greater radical-scavenging capacity than the fruit preparations most commonly investigated in the literature.

A particularly analytically significant observation was the exceptional CUPRAC (1065.85 mg TE per g) and FRAP (992.34 mg TE per g) values recorded for *R. horrida* leaf water extract, the highest reducing power values in the entire dataset, exceeding even the corresponding MeOH extract of the same species in these two assays. The results can be explained by the high content of anthocyanins and flavonols, which perform well in electron-transfer-based reducing assays due to their multiple redox-active groups, which undergo rapid and complete electron donation under CUPRAC and FRAP conditions.^40,45^

EA extracts showed markedly lower radical scavenging capacity compared to polar extracts, most strikingly in leaf EA samples (DPPH: 23.43–228.80 mg TE per g). This dissociation between polarity and radical-scavenging activity is mechanistically consistent with the compositional profile of EA extracts: aglycone flavonoids and lipophilic phenolic esters, which predominate in this polarity window, are less efficient hydrogen-atom donors in the aqueous DPPH and ABTS assays compared to the water-soluble polyphenols dominating MeOH and water extracts.^38,39,44^ This observation does not diminish the pharmacological relevance of EA extracts; rather, it reflects the well-established principle that radical-scavenging capacity and target-specific bioactivity represent distinct and not necessarily correlated pharmacological properties.^38,39,44^

MCA values revealed a markedly different solvent–activity relationship from the radical-scavenging assays: water extracts consistently achieved the highest metal chelating activity across all species (19.95–25.04 mg EDTAE per g), substantially exceeding both MeOH and EA extracts. This pattern reflects the structural prerequisites for effective metal chelation, *ortho*-dihydroxyl groups and free carboxylate moieties, being most abundantly represented in water-soluble phenolic acids and hydrolysable tannins.^42^*R. hemisphaerica* leaf water extract recorded the highest MCA in the entire dataset (25.04 mg EDTAE per g), while its EA counterpart showed the lowest (9.28 mg EDTAE per g), a nearly three-fold difference consistent with complete phase separation of metal-chelating polar phenolics into the aqueous fraction. By sequestering redox-active Fe^2+^ and Cu^2+^ ions, these water-soluble extracts suppress Fenton-type radical generation, a mechanistically distinct antioxidant pathway complementary to direct radical scavenging.^42^ PPM values (2.48–5.06 mmol TE per g), reflecting total reductive capacity by phosphomolybdenum reduction,^30^ corroborated FRAP and CUPRAC trends, with MeOH leaf extracts uniformly achieving the highest values (*R. canina*: 5.03; *R. hemisphaerica*: 5.06 mmol TE per g), consistent with the elevated TPC and flavonoid content of these extracts.

Taken together, the antioxidant findings suggest that all four Turkish *Rosa* species possess substantial and mechanistically diverse antioxidant capacities, with the vegetative organs of the three understudied species, *R. hemisphaerica*, *R. horrida*, and *R. pulverulenta*, fully comparable to or exceeding *R. canina* under identical conditions. Furthermore, solvent polarity is the dominant determinant of activity magnitude across most assays. MeOH and water extracts excel in radical scavenging and metal chelation, respectively, while EA extracts display lower but structurally informative activity profiles. Third, the exceptional CUPRAC and FRAP values of *R. horrida* leaf water extract identify this fraction as particularly enriched in electron-donating constituents, most likely ellagitannins, representing a high-value matrix for targeted isolation of specific reducing-type antioxidants.

### 3.3. Enzyme inhibitory effects

#### 3.3.1. Cholinesterase inhibition

The enzyme inhibitory activities of all 24 extracts are presented in Table 5. Acetylcholinesterase (AChE) and butyrylcholinesterase (BChE) are the primary pharmacological targets for Alzheimer's disease (AD) therapy: inhibition of these enzymes prolongs the synaptic availability of acetylcholine and, in the case of BChE, compensates for the progressive AChE deficit characteristic of advanced disease.^46^ The highest AChE inhibition was recorded for *R. horrida* leaves EA (2.49 mg GALAE per g) and *R. pulverulenta* twigs EA (2.42 mg GALAE per g). Of all the extracts evaluated, only one (*R. hemispharerica* twigs-EA) showed no activity against AChE. These values are competitive with those reported for other *Rosa* species in comparative enzyme inhibition studies: Findik *et al.* (2024) documented significant AChE inhibitory activity for *R. pimpinellifolia* fruit extracts,^12^ and Sağlamtas *et al.* (2025) reported AChE inhibition with IC_50_ 62.27 µg mL^−1^ for *R. pisiformis* ethanol extract.^11^ The mechanistic basis for cholinesterase inhibition by polyphenols is well characterized: flavonoid aglycones, particularly quercetin, luteolin, and kaempferol, interact with both the catalytic anionic site (CAS) and peripheral anionic site (PAS) of AChE *via* π-stacking with aromatic residues, hydrogen bonding with active site amino acids, and hydrophobic contacts along the gorge lining.^47^

**Table 5:** Enzyme inhibitory effects of the tested extracts^a^

*Rosa* species	Parts	Extracts	AChE (mg GALAE per g)^b^	BChE (mg GALAE per g)^b^	Tyrosinase (mg KAE per g)^c^	Amylase (mmol ACAE per g)^d^	Glucosidase (mmol ACAE per g)^d^
*Rosa canina*	Twigs	EA	2.29 ± 0.02^bc^	4.24 ± 0.17^ab^	50.31 ± 0.42^ef^	0.63 ± 0.01^c^	0.17 ± 0.01^c^
		MeOH	2.42 ± 0.04^ab^	4.01 ± 0.03^bcd^	70.05 ± 0.20^ab^	0.52 ± 0.02^ef^	0.19 ± 0.01^a^
		Water	1.69 ± 0.02^f^	1.11 ± 0.22^g^	43.01 ± 0.47^ghij^	0.06 ± 0.01^i^	NA
	Leaves	EA	2.01 ± 0.01^d^	NA	43.14 ± 0.71^fghij^	0.56 ± 0.01^de^	0.18 ± 0.01^ab^
		MeOH	2.35 ± 0.01^abc^	3.47 ± 0.03^e^	73.14 ± 0.18^a^	0.49 ± 0.01^f^	NA
		Water	1.83 ± 0.04^ef^	0.67 ± 0.09^h^	36.06 ± 1.13^jk^	0.06 ± 0.01^i^	NA
*Rosa hemisphaerica*	Twigs	EA	NA	NA	54.00 ± 1.96^de^	0.70 ± 0.02^ab^	0.18 ± 0.01^ab^
		MeOH	2.42 ± 0.01^ab^	4.16 ± 0.04^bc^	60.36 ± 0.51^cd^	0.39 ± 0.03^g^	0.18 ± 0.01^ab^
		Water	1.79 ± 0.03^ef^	1.21 ± 0.16^g^	39.85 ± 0.51^ghij^	0.06 ± 0.01^i^	NA
	Leaves	EA	1.86 ± 0.12^de^	NA	36.68 ± 0.89^ijk^	0.57 ± 0.02^d^	0.07 ± 0.01^e^
		MeOH	2.25 ± 0.07^c^	3.52 ± 0.05^e^	59.75 ± 0.61^cd^	0.43 ± 0.01^g^	NA
		Water	1.77 ± 0.04^ef^	0.71 ± 0.11^h^	29.64 ± 1.02^kl^	0.16 ± 0.01^h^	NA
*Rosa horrida*	Twigs	EA	2.34 ± 0.04^abc^	4.02 ± 0.08^bcd^	63.58 ± 8.92^bc^	0.72 ± 0.01^a^	0.15 ± 0.01^d^
		MeOH	2.40 ± 0.02^abc^	4.02 ± 0.05^bcd^	69.67 ± 0.23^ab^	0.52 ± 0.01^ef^	0.19 ± 0.01^a^
		Water	1.46 ± 0.06^g^	0.17 ± 0.02^i^	44.96 ± 0.43^fg^	0.06 ± 0.01^i^	NA
	Leaves	EA	2.49 ± 0.03^a^	3.91 ± 0.10^cd^	39.87 ± 1.76^ghij^	0.55 ± 0.01^de^	NA
		MeOH	2.34 ± 0.01^abc^	3.35 ± 0.05^ef^	72.97 ± 0.68^a^	0.49 ± 0.01^f^	0.18 ± 0.01^ab^
		Water	1.73 ± 0.07^ef^	NA	42.50 ± 0.75^ghij^	0.06 ± 0.01^i^	NA
*Rosa pulverulenta*	Twigs	EA	2.42 ± 0.03^ab^	3.52 ± 0.09^e^	43.81 ± 5.32^fghi^	0.72 ± 0.01^a^	0.15 ± 0.01^d^
		MeOH	2.39 ± 0.05^abc^	3.82 ± 0.09^d^	68.72 ± 0.56^ab^	0.49 ± 0.01^f^	0.18 ± 0.01^ab^
		Water	1.30 ± 0.04^g^	NA	44.11 ± 0.83^fgh^	0.07 ± 0.01^i^	NA
	Leaves	EA	2.26 ± 0.13^bc^	4.44 ± 0.09^a^	37.40 ± 1.87^hij^	0.66 ± 0.03^bc^	0.17 ± 0.01^bc^
		MeOH	2.28 ± 0.02^bc^	3.15 ± 0.08^f^	66.94 ± 0.20^abc^	0.41 ± 0.01^g^	0.19 ± 0.01^a^
		Water	1.43 ± 0.05^g^	NA	26.66 ± 1.44^l^	0.04 ± 0.01^i^	NA

a Values are means ± SD of three measurements. Different letters indicate significant differences between the tested extracts (*p* < 0.05).

b mg Galantamine equivalents per g of extract.

c mg Kojic acid per g of extract.

d mmol Acarbose equivalents per g of extract.

BChE inhibition showed a markedly more selective distribution: water extracts and several leaf EA extracts yielded no detectable activity, while twig-derived EA and MeOH extracts exhibited moderate but consistent inhibition (3.15–4.44 mg GALAE per g). The highest BChE inhibition was observed for *R. pulverulenta* leaves EA (4.44 mg GALAE per g), exceeding *R. canina* twigs EA (4.24 mg GALAE per g). This differential distribution, BChE activity confined to lipophilic extracts, AChE activity more broadly distributed, is consistent with the known structural preference of BChE for bulkier, more lipophilic substrates and inhibitors, arising from the larger and less constrained active site gorge of BChE relative to AChE.^46^ This selectivity has direct therapeutic relevance: BChE-selective inhibitors are of particular interest for late-stage AD management, where AChE-targeted therapy progressively loses efficacy as AChE levels decline and BChE increasingly assumes acetylcholine hydrolysis.^46^ The identification of twig EA extracts from all four Turkish *Rosa* species as BChE-active represents a novel finding, with no precedent in the literature for any of the four species studied.

#### 3.3.2. Tyrosinase inhibition

All 24 extracts exhibited significant tyrosinase inhibitory activity, with MeOH extracts consistently achieving the highest values (59.75–73.14 mg KAE per g for leaf extracts across all species). Tyrosinase is a binuclear copper-containing enzyme that catalyzes the rate-limiting hydroxylation of l-tyrosine to l-DOPA and subsequent oxidation to dopaquinone, the committed step in melanin biosynthesis; its inhibition is a primary therapeutic and cosmeceutical strategy for hyperpigmentation disorders, including melasma, post-inflammatory hyperpigmentation, and melanoma progression.^48^ The high tyrosinase inhibitory capacity of all four Turkish *Rosa* species is consistent with the well-established inhibitory activity of their constituent phenolics: gallic acid, ellagic acid, and flavonols, particularly quercetin and kaempferol, are potent tyrosinase inhibitors that interact with the catalytic copper center *via* chelation and competitive displacement of the substrate.^14,48^ The EA extracts also showed substantial activity (36.68–63.58 mg KAE per g), with *R. horrida* twigs EA (63.58 mg KAE per g) yielding the highest EA-fraction value in the dataset, a result consistent with the high flavonoid content of this extract (Table 1). Water extracts showed lower but pharmacologically relevant inhibition (26.66–44.96 mg KAE per g). The tyrosinase inhibitory activity reported here for the leaf and twig extracts of *R. hemisphaerica*, *R. horrida*, and *R. pulverulenta* substantially exceeds values published for *R. canina* rosehip extracts in the available literature^14^ and identifies vegetative organs of these understudied species as promising sources for the isolation of specific tyrosinase inhibitors relevant to cosmeceutical and dermatological applications.

#### 3.3.3. α-Amylase and α-glucosidase inhibition

Inhibition of pancreatic α-amylase and intestinal α-glucosidase is the mechanism of action of the antidiabetic drug acarbose and represents a validated strategy for attenuating postprandial hyperglycemia in type 2 diabetes mellitus by retarding starch hydrolysis and monosaccharide absorption.^13^ EA extracts exhibited the highest α-amylase inhibitory activity across all species and organs: twig EA extracts ranged from 0.57 to 0.72 mmol ACAE per g and leaf EA extracts from 0.55 to 0.66 mmol ACAE per g, while water extracts were minimally active (0.04–0.07 mmol ACAE per g). This pronounced lipophilicity-activity dependence is consistent with the preferential inhibition of mammalian α-amylase by aglycone flavonoids and lipophilic terpenoids such as ursolic acid and oleanolic acid, rather than their more abundant glycosidic counterparts, which display substantially weaker amylase-binding affinity.^13^ Among individual fractions, *R. pulverulenta* twigs EA (0.72 mmol ACAE per g) and *R. hemisphaerica* twigs EA (0.70 mmol ACAE per g) recorded the highest values, suggesting that these species may harbor structurally specific lipophilic amylase inhibitors warranting targeted isolation efforts.

α-Glucosidase inhibitory activity was mainly observed in the twig EA extracts (0.15–0.19 mmol ACAE per g). The EA and MOH fractions of the leaf extracts generally showed a glucosidase inhibition, whereas the aqueous extracts exhibited no detectable activity. This strong organ- and polarity-specificity, twig EA extracts consistently more active than all other fractions, parallels the elevated TPC and TFC values of twig EA extracts documented in Section 3.1 and reflects the likely concentration of specific aglycone inhibitors in the bark matrix. The α-glucosidase inhibitory activity of *R. canina* twig EA (0.17–0.19 mmol ACAE per g) is reported here for the first time for a twig-derived extract; published *R. canina* glucosidase inhibition data have previously been confined to rosehip preparations.^8,9^ Across all four species, the organ-specific and polarity-restricted pattern of both α-amylase and α-glucosidase inhibition is internally consistent and mechanistically coherent: the selective accumulation of lipophilic aglycones in twig bark, documented compositionally in Section 3.1, translates directly into selective starch-processing enzyme inhibition in this extract, providing a structurally plausible basis for the observed activity profile.

#### 3.3.4. Carbonic anhydrase inhibition

The CA I and CA II inhibitory activities of all 24 extracts (IC_50_, µg mL^−1^; acetazolamide as standard: CA I IC_50_ = 3.83 ng mL^−1^; CA II IC_50_ = 2.48 ng mL^−1^) are presented in Table 6. This represents the first evaluation of carbonic anhydrase inhibition by any of the four studied *Rosa* species. Carbonic anhydrases are zinc metalloenzymes that catalyze the reversible hydration of CO_2_ to bicarbonate and are validated drug targets for glaucoma, diuresis, epilepsy, and, through the tumor-associated isoforms CA IX and CA XII, for oncological applications.^49^ As expected for complex polyphenolic plant extracts relative to a targeted synthetic sulfonamide, IC_50_ values were substantially higher than the acetazolamide reference; nevertheless, the EA extracts demonstrated dramatically superior potency relative to the corresponding MeOH and water extracts, with IC_50_ values for EA extracts ranging from 12.17 to 25.29 µg mL^−1^ (CA I) and 15.36 to 28.87 µg mL^−1^ (CA II).

**Table 6:** Carbonic anhydrase (CAI and CAII) inhibitory effects of the tested extracts^a^

*Rosa* species	Parts	Extracts	CA I		CA II	
			IC_50_ (µg mL^−1^)	*R* ^2^	IC_50_ (µg mL^−1^)	*R* ^2^
*Rosa canina*	Twigs	EA	13.58 ± 0.10^bc^	0.9925	26.65 ± 0.57^de^	0.9785
		MeOH	91.18 ± 0.24^j^	0.9974	83.49 ± 2.45^i^	0.9706
		Water	92.40 ± 0.08^j^	0.9991	110.00 ± 4.84^k^	0.9560
	Leaves	EA	14.80 ± 0.02^c^	0.9989	21.52 ± 0.01^cd^	0.9998
		MeOH	18.23 ± 0.12^de^	0.9932	13.69 ± 0.01^b^	0.9997
		Water	147.44 ± 0.32^n^	0.9978	99.00 ± 0.54^j^	0.9945
*Rosa hemisphaerica*	Twigs	EA	25.29 ± 0.62^f^	0.9754	17.50 ± 0.03^bc^	0.9981
		MeOH	97.60 ± 2.31^k^	0.9763	101.91 ± 1.21^j^	0.9881
		Water	93.64 ± 0.06^j^	0.9994	80.58 ± 0.16^i^	0.9980
	Leaves	EA	15.43 ± 0.14^c^	0.9910	28.87 ± 1.28^c^	0.9557
		MeOH	82.50 ± 0.01^i^	0.9999	119.48 ± 1.53^l^	0.9872
		Water	123.75 ± 0.20^m^	0.9984	121.57 ± 0.63^l^	0.9948
*Rosa horrida*	Twigs	EA	15.67 ± 0.02^c^	0.9987	15.85 ± 1.11^b^	0.9300
		MeOH	71.44 ± 1.64^h^	0.9771	84.51 ± 0.15^i^	0.9982
		Water	54.14 ± 0.53^g^	0.9903	56.80 ± 2.85^g^	0.9499
	Leaves	EA	15.82 ± 0.03^cd^	0.9979	15.36 ± 0.07^b^	0.9955
		MeOH	103.43 ± 0.87^l^	0.9916	108.28 ± 0.26^k^	0.9976
		Water	52.90 ± 1.27^g^	0.9760	39.82 ± 1.41^f^	0.9647
*Rosa pulverulenta*	Twigs	EA	15.60 ± 0.12^c^	0.9920	16.42 ± 0.07^bc^	0.9958
		MeOH	69.30 ± 2.11^h^	0.9695	72.18 ± 4.61^h^	0.9361
		Water	126.00 ± 0.18^m^	0.9986	85.55 ± 0.53^i^	0.9938
	Leaves	EA	12.17 ± 0.08^b^	0.9936	26.86 ± 0.73^de^	0.9729
		MeOH	20.08 ± 0.02^e^	0.9992	24.31 ± 0.18^de^	0.9924
		Water	96.25 ± 0.72^k^	0.9915	100.43 ± 2.16^j^	0.9896
Asetazolamid (standart inhibitor) (ng mL^−1^)			3.83 ± 0.04^a^	0.9900	2.48 ± 0.07^a^	0.9705

a Values are means ± SD of three measurements. Different letters indicate significant differences between the tested extracts (*p* < 0.05).

The most potent CA inhibitors were: *R. pulverulenta* leaves EA (CA I IC_50_ = 12.17 µg mL^−1^; CA II IC_50_ = 26.86 µg mL^−1^), *R. canina* twigs EA (CA I IC_50_ = 13.58 µg mL^−1^), *R. horrida* leaves EA (CA I IC_50_ = 15.82 µg mL^−1^; CA II IC_50_ = 15.36 µg mL^−1^), and *R. canina* leaves MeOH (CA I IC_50_ = 18.23 µg mL^−1^; CA II IC_50_ = 13.69 µg mL^−1^). The mechanistic basis for CA inhibition by polyphenols is well established: *ortho*-dihydroxyl groups on aromatic rings can coordinate the catalytic zinc ion at the CA active site, either directly or *via* displacement of the zinc-bound water molecule, while the aromatic scaffold contributes additional van der Waals contacts and π-stacking interactions with hydrophobic active site residues.^50,51^ Gallic acid, ellagic acid, quercetin, and caffeic acid, all characteristic constituents of *Rosa* EA extracts, have been individually confirmed as effective CA I and CA II inhibitors with Ki values in the low micromolar to submicromolar range.^51^

By contrast, MeOH and water extracts showed substantially reduced CA inhibitory potency (IC_50_ typically 52–147 µg mL^−1^), consistent with the poor structural complementarity between glycosylated polyphenols and the relatively narrow, hydrophobic CA active site gorge: the bulky sugar moieties of flavonoid glycosides sterically hinder zinc coordination and reduce the planarity required for productive active site binding.^50,51^ This pattern, EA extracts selectively active, polar extracts substantially weaker, mirrors precisely the selectivity observed for tyrosinase and cholinesterase inhibition in Sections 3.3.1 and 3.3.2, reinforcing the conclusion that a specific lipophilic aglycone-enriched phenolic subclass constitutes the pharmacologically dominant component of the EA extract across multiple enzyme targets. The selectivity of EA extracts for CA inhibition, combined with their established structural enrichment in aglycone flavonoids documented in Section 3.1, provides a compositionally coherent and mechanistically plausible basis for the observed activity profile and identifies these extracts as priority candidates for targeted isolation of individual CA-inhibitory constituents.

### 3.4. Cytotoxicity

The initial screening was conducted using two cell lines, non-cancerous VERO cells and H1HeLa cells derived from cervical adenocarcinoma. Results of cytotoxicity testing were evaluated in accordance with the classification system for plant extract cytotoxicity as proposed by the National Cancer Institute^52^ and previously published work.^25^ Screening results (Table 7) indicated that ethyl acetate extracts from all tested *Rosa* species exhibited moderate cytotoxicity (CC_50_ values ranging from 28.04 to 63.10 µg mL^−1^) and significant selectivity (SI values ranging from 4.65 to 7.69) towards cancer-derived cells. Thus, ethyl acetate extracts were selected for further testing using MDA-MB-231 (breast adenocarcinoma) and A375 (malignant melanoma) cells. The results indicated high cytotoxicity (CC_50_ < 20 µg mL^−1^) of EA extracts from most *Rosa* species against A375 malignant melanoma cells (Table 8). Overall, for A375 cells, extracts from twigs were more cytotoxic than those from leaves. The highest cytotoxicity was observed in EA extracts from *R. hemisphaerica* twigs and leaves, with CC_50_ values of 3.22 and 8.85 µg mL^−1^, respectively. Importantly, the *R. hemisphaerica* twig EA extract showed highly selective cytotoxic activity against A375 cells, with an SI of 73.95, compared to VERO cells.

**Table 7:** Cytotoxicity screening using two cell lines – normal and cancer-derived

*Rosa* species	Parts	Extracts	CC_50_ ± SD (µg mL^−1^)^a^		SI^b^
			VERO	H1HeLa	
*Rosa canina*	Twigs	EA	448.15 ± 3.18	58.26 ± 1.04	**7.69**
		MeOH	156.75 ± 1.48	335.45 ± 21.43	0.47
		Water	158.00 ± 10.32	469.45 ± 3.61	0.34
	Leaves	EA	362.25 ± 21.85	63.10 ± 6.53	**5.74**
		MeOH	103.78 ± 11.21	195.60 ± 9.90	0.53
		Water	183.95 ± 2.19	288.65 ± 19.59	0.64
*Rosa hemispherica*	Twigs	EA	238.45 ± 6.86	50.35 ± 4.31	**4.74**
		MeOH	470.30 ± 4.38	226.05 ± 13.93	2.08
		Water	261.95 ± 20.15	423.30 ± 17.25	0.62
	Leaves	EA	243.90 ± 2.69	53.32 ± 2.17	**4.57**
		MeOH	178.55 ± 10.25	125.80 ± 8.06	1.42
		Water	162.70 ± 8.34	230.65 ± 3.46	0.71
*Rosa horrida*	Twigs	EA	430.35 ± 13.36	56.40 ± 2.12	**7.63**
		MeOH	258.85 ± 9.69	170.45 ± 7.71	1.52
		Water	476.30 ± 28.00	408.60 ± 1.27	1.17
	Leaves	EA	140.60 ± 7.07	30.21 ± 0.97	**4.65**
		MeOH	94.74 ± 5.37	104.90 ± 6.65	0.90
		Water	116.35 ± 9.40	269.75 ± 6.15	0.43
*Rosa pulverulenta*	Twigs	EA	220.15 ± 5.87	28.15 ± 0.27	**7.82**
		MeOH	321.75 ± 7.28	234.30 ± 2.97	1.37
		Water	254.10 ± 7.07	304.25 ± 9.97	0.84
	Leaves	EA	150.25 ± 0.21	28.04 ± 0.70	**5.36**
		MeOH	221.05 ± 2.62	145.30 ± 3.11	1.52
		Water	257.00 ± 1.41	376.90 ± 5.94	0.68

a CC_50_ – 50% cytotoxic concentration (µg mL^−1^); mean ± SD.

b SI – selectivity index (VERO CC_50_/H1HeLa CC_50_).

**Table 8:** Cytotoxicity testing of selected extracts using additional cancer-derived cell lines

*Rosa* species	Part/extract	MDA MB 231		A375	
		CC_50_ ± SD^a^	SI^b^	CC_50_ ± SD	SI
*Rosa canina*	Twigs-EA	73.38 ± 2.67	6.11	19.91 ± 0.66	22.51
	Leaves-EA	90.43 ± 4.89	4.01	28.08 ± 1.38	12.90
*Rosa hemispherica*	Twigs-EA	50.16 ± 1.34	4.75	3.22 ± 0.20	73.95
	Leaves-EA	52.72 ± 1.45	4.63	8.85 ± 0.53	27.55
*Rosa horrida*	Twigs-EA	94.05 ± 2.36	4.58	16.11 ± 1.36	26.71
	Leaves-EA	41.72 ± 2.79	3.37	22.85 ± 0.57	6.15
*Rosa pulverulenta*	Twigs-EA	75.48 ± 4.49	2.92	17.79 ± 1.00	12.37
	Leaves-EA	71.02 ± 2.85	2.12	20.83 ± 0.84	7.21

a CC_50_ – 50% cytotoxic concentration (µg mL^−1^); mean ± SD.

b SI – selectivity index (VERO CC_50_/cancer cell line CC_50_). The CC_50_ values for VERO cells are presented in Table 6.

There are already several reports on the cytotoxicity of different *Rosa* species. In our research, the *R. hemisphaerica* twig EA extract exhibited the highest cytotoxicity against cancer-derived cells among the four species tested, while Dehghan Kashani *et al.*^53^ reported that the aqueous extract of *R. hemisphaerica* flowers originating from Iran exhibited weak cytotoxicity against HeLa cells (CC_50_ of 327 µg mL^−1^) and was moderately cytotoxic to human lymphocytes (CC_50_ of 177 µg mL^−1^) after 48 h incubation. However, the *Rosa damascena* flower aqueous extract was highly cytotoxic to HeLa cells (CC_50_ of 4.5 µg mL^−1^) and moderately cytotoxic to human lymphocytes (CC_50_ of 115.7 µg mL^−1^).^54^ Conversely, a commercially available aqueous-alcoholic *R. damascena* extract showed weak to no cytotoxic effects on both human diploid fibroblasts (HSF) and the human hepatocarcinoma cell line (HepG2).^55^ Al-Oqail *et al.*^56^ reported no cytotoxicity of hexane and methanolic extracts of *R. damascena* flowers against MCF-7 (human breast adenocarcinoma) and A549 (human lung cancer) cells. In the case of HeLa cells, the hexane extract was not cytotoxic, but the methanolic extract exerted moderate cytotoxicity (198.4 µg mL^−1^). It was also found that the methanolic extract induced apoptosis in HeLa cells, and reported that both the pro-apoptotic effect and the resulting cytotoxicity in HeLa cells might be linked to the induction of oxidative stress, ROS formation, mitochondrial depolarisation, and cell cycle arrest.^56^ Extracts from *R. canina* obtained using acidified DMSO (1% acetic acid) showed a cytotoxic effect on A549 and PC-3 (human prostate cancer) cells with a CC_50_ of 142.5 and 209.6 µg mL^−1^, respectively, but with relatively low selectivity (SI 1.6–2.4) compared to normal human foreskin fibroblast cells.^57^ A similarly low selectivity (SI = 1.5) was observed when cytotoxicity was assessed in WiDr (human colon adenocarcinoma) and human normal colon epithelial cell lines (CCD 841 CoN).^58^ Despite low selectivity, extracts were shown to induce cell-cycle arrest and apoptosis *via* reduced mitochondrial membrane potential (MMP) and increased caspase activity,^57,58^ as well as to repress telomerase expression.^58^ It can be observed that the cytotoxicity of *Rosa* species depends not only on the studied plant species and cell line panel but also on the extractant and the plant part used for extraction. Importantly, it should be emphasized that the novelty of our cytotoxicity study lies not only in the applied screening procedure and the selection of the cell line panel, but also in the focus on previously understudied parts of those *Rosa* species – twigs and leaves.

### 3.5. The correlation between phytochemical parameters and bioactivity assays

**Fig. 3:**
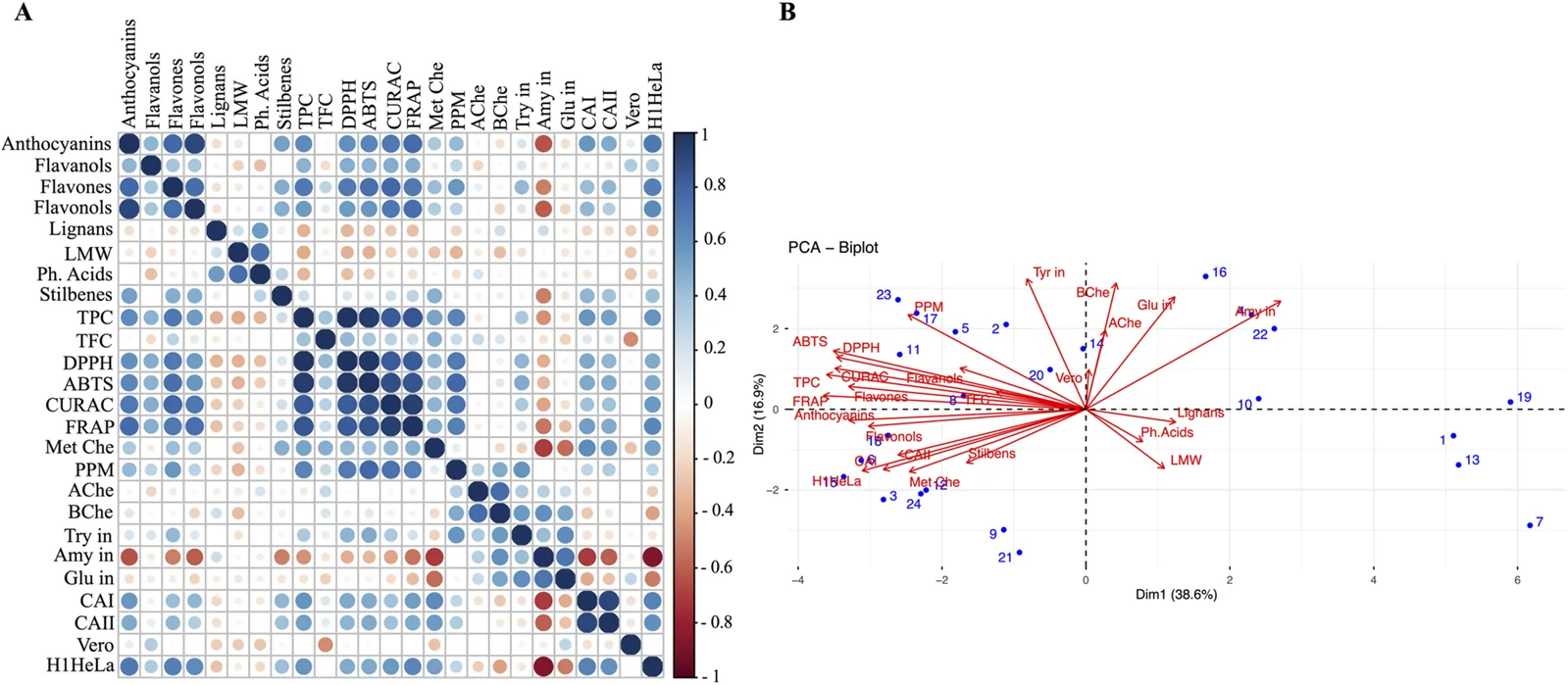
(A) Pearson correlation matrix showing the relationship between phenolic subclasses, bioassays, and cytotoxicity parameters. (B) Principal component analysis (PCA) biplot showing the distribution of all parameters in all samples. Arrows indicate the contribution and direction of each variable to the principal components. Legend: 1: *R. canina*, Leaf, EA, 2: *R. canina*, Leaf, MeOH, 3: *R. canina*, Leaf, Water, 4: *R. canina*, Twig, EA, 5: *R. canina*, Twig, MeOH, 6: *R. canina*, Twig, Water, 7: *R. hemispherica*, Leaf, EA, 8: *R. hemispherica*, Leaf, MeOH, 9: *R. hemispherica*, Leaf, Water, 10: *R. hemispherica*, Twig, EA, 11: *R. hemispherica*, Twig, MeOH, 12: *R. hemispherica*, Twig, Water, 13: *R. horrida*, Leaf, EA, 14: *R. horrida*, Leaf, MeOH, 15: *R. horrida*, Leaf, Water, 16: *R. horrida*, Twig, EA, 17: *R. horrida*, Twig, MeOH, 18: *R. horrida*, Twig, Water, 19: *R. pulverulenta*, Leaf, EA, 20: *R. pulverulenta*, Leaf, MeOH, 21: *R. pulverulenta*, Leaf, Water, 22: *R. pulverulenta*, Twig, EA, 23: *R. pulverulenta*, Twig, MeOH, 24: *R. pulverulenta*, Twig, Water.

The correlation among parameters and their distribution were displayed using the heatmap and PCA analyses (Fig. 3). The Pearson correlation analysis (Fig. 3A, Table S3) showed that TPC, TFC, and the four antioxidant assays (DPPH, ABTS, CUPRAC, and FRAP) form a tightly clustered block of strong, consistent positive correlations (*r* = 0.85–0.98). The enzyme–inhibition parameters AChE and BChE correlated strongly with each other, but only weakly with the antioxidant assays. In contrast, inhibition of tyrosinase, amylase, and glucosidase showed a more mixed pattern. Moderate correlation was observed between carbonic anhydrase inhibition (CAI/CAII) and the flavonoid-antioxidant cluster. This suggests that flavonoid-rich extracts may contribute more significantly to carbonic anhydrase inhibitory activity. The cytotoxicity endpoints (Vero, H1, and HeLa) clustered together and predominantly showed negative correlations with the phenolic/antioxidant block, most notably with the cancer cell lines (H1 and HeLa).

The PCA biplot resolved 55.5% of the total variance across the first two dimensions (Dim1 = 38.6%; Dim2 = 16.9%). Dim1 separated total phenolic/flavonoid content and antioxidant assays (TPC, TFC, DPPH, ABTS, CUPRAC, and FRAP), along with anthocyanins, flavonols, flavanols, CAI/CAII, metal chelating, and H1/HeLa cytotoxicity on its negative side. On the positive side, EA extracts clustered, associated with lignans, low-molecular-weight polyphenols, and phenolic acids, and assessed amylase inhibition, confirming the inverse relationship between these groups, as already suggested by the correlation heatmap. Dim2 largely captured cholinesterase-related activity, with AChE, BChE, PPM, and tyrosinase inhibition loading strongly and positively independently of the phenolic/antioxidant axis. Vero cytotoxicity loaded weakly on both dimensions, plotting near the origin. Overlaying the sample identities onto this structure suggests that the extraction solvent, rather than the species, drove much of the separation. Water extracts (*R. hemisphaerica* leaf and twig, and *R. pulverulenta* leaf) clustered in the strongly negative Dim2 region. Methanol extracts (*R. horrida* twig and *R. hemisphaerica* twig) fell in the positive Dim2/negative Dim1 region. Several ethyl acetate extracts (*R. pulverulenta* leaf, *R. horrida* leaf, and *R. hemisphaerica* twig) plotted towards positive Dim1. This indicated that methanol and ethyl acetate extracts tend to exhibit greater enzyme inhibitory or lignan/phenolic acid activity, whereas water extracts are compositionally distinct and comparatively low on both the antioxidant/flavonoid and enzyme inhibition axes.

## 4 Conclusion

This study provides the first comprehensive phytochemical and multi-target bioactivity evaluation of the vegetative organs of four Turkish *Rosa* species, including the first data of any kind for *R. hemisphaerica*, *R. horrida*, and *R. pulverulenta*. Three principal conclusions emerge from the dataset. Vegetative organs are phenolically superior to the fruit preparations that dominate the *Rosa* literature: MeOH and water TPC values (≥97 mg GAE per g) exceeded published rosehip fruit data by 4- to 10-fold, and leaf MeOH TFC (55–58 mg RE per g) surpassed fruit-derived values by 14- to 90-fold. The untargeted metabolomics analysis resolved 337 phenolic compounds, with flavonoids accounting for nearly two-thirds of the annotated chemical profile. HCA confirmed a clean separation between EA and polar fractions that was consistent across all four species, which was supported by the AMOPLS decomposition, showing that solvent polarity was the dominant driver of compositional variation (28.6%), outweighing species and tissue effects. In addition, tissue-level partitioning was equally consistent: twig tissue concentrated flavonoid subclasses, including anthocyanins, flavanols, flavones, and flavonols, while leaf tissue was enriched in lignans, phenolic acids, and LMW polyphenols.

The twig EA fraction emerged as a pharmacologically privileged matrix, displaying unexpectedly high TPC (83–105 mg GAE per g) and TFC (35–47 mg RE per g) alongside convergent selective activity across all enzyme targets, cholinesterase, tyrosinase, α-amylase, α-glucosidase, and carbonic anhydrase, consistent with selective enrichment in aglycone flavonoids. The *in vitro* cytotoxicity studies revealed that ethyl acetate extracts of *Rosa hemisphaerica* twigs and leaves exhibit high cytotoxicity against the A375 cell line derived from malignant melanoma, with low CC_50_ values (3.22–8.85 µg mL^−1^) and selectivity (SI 27.55–73.95) compared to non-cancerous cells. The three previously unstudied species are fully comparable to or exceed *R. canina* across all measured parameters; notably, the BChE-selective inhibitory activity of twig EA fractions, reported here for the first time, is of direct relevance for late-stage Alzheimer's disease management, and the exceptional reducing power of *R. horrida* leaf water extract warrants targeted investigation of its ellagitannin composition. Collectively, these findings quantitatively demonstrate that Turkish *Rosa* vegetative organs, and twig EA fractions in particular, represent multi-target bioactive matrices whose pharmacological potential has been systematically underestimated, and provide a scientifically grounded basis for future bioactivity-guided isolation and *in vivo* validation studies.

## Author contributions

Hajar Salehi: investigation, methodology, formal analysis, data curation, writing – original draft, writing – review and editing. Milena Terzic: investigation, methodology, data curation, writing – original draft, writing – review and editing. Gunes Ak: investigation, methodology, writing – original draft, writing – review and editing. Gokhan Zengin: investigation, methodology, data curation, writing – original draft, writing – review and editing, supervision, project administration. Łukasz Świątek: methodology, writing – original draft, writing – review and editing. Kinga Salwa: methodology, writing – original draft, writing – review and editing. Ismail Yapıcı: methodology, writing – original draft, writing – review and editing. Ilhami Gulcin: investigation, methodology, data curation, writing – original draft, writing – review and editing, supervision. Evren Yildiztugay: investigation, methodology, resources, writing – original draft. Luigi Lucini: writing – review and editing, funding acquisition, resources, supervision.

## Conflicts of interest

The authors declare no competing interests.

## Supplementary Material

RA-OLF-D6RA07053A-s001

## Data Availability

The data that support the findings of this study are available on request from the corresponding author. Supplementary information (SI): Table S1. The putatively annotated compounds in the extracts. Table S2. VIP scores for identified compounds using the AMOPLS decomposition. Table S3. The Pearson correlation analysis. See DOI: https://doi.org/10.1039/d6ra07053a.
